# Cardiovascular endpoints and psychosocial challenges of lipoprotein(a) of 5726 participants in the ELITE-study over 5 years

**DOI:** 10.1186/s12944-025-02785-2

**Published:** 2025-11-14

**Authors:** Bastian Schrader, Friedrich Lorenz, Armin Weers, Matteo Scorcelletti, Stephan Lüders, Bernhard Vaske, Sandra Garstecki, Joachim Schrader, Albrecht Elsässer

**Affiliations:** 1 Department of Cardiology, University of Oldenburg, Klinikum Oldenburg, Oldenburg, Germany; 2 Department of Nephrology, Internal Medicine, St.-Josefs-Hospital, Cloppenburg, Germany; 3 Institute for Hypertension and Cardiovascular Research (INFO), Cloppenburg, Germany; 4 Department of cardiology-University Oldenburg- Klinikum Oldenburg, Oldenburg, 26133 Germany

**Keywords:** Lipoprotein(a), Cardiovascular risk, Preventive medicine, Psychosocial burden, Lipid management

## Abstract

**Background:**

Lipoprotein(a) [Lp(a)] is a known independent risk factor for cardiovascular disease, yet awareness and management remain limited. The psychosocial implications of elevated Lp(a)-levels have been poorly characterized.

**Objectives:**

To compare cardiovascular outcomes and cardiovascular risk factor (CVRF) modification in individuals with normal vs. elevated Lp(a) levels, and to assess the impact of individualized prevention recommendations. For the first time, the individual psychological stress caused by Lp(a) is being surveyed.

**Methods:**

The ELITE study is a prospective, interventional cohort study conducted in north-western Germany. Participants were regularly assessed for CVRFs, including Lp(a), hypertension, dyslipidemia, diabetes mellitus, weight, nicotine – as well as lipoprotein (a), physical activity, dietary habits, depression and stress. They received written, personalized prevention recommendations. Follow-up averaged 4.4 years. Two groups were analyzed: Group 1 (Gr1, n=3,241) with normal Lp(a), and Group 2 (Gr2, n=841) with elevated Lp(a ≥75 nmol/l).

**Results:**

Gr2 (mean Lp(a) 154.8 nmol/l) and Gr1 (mean Lp(a) 16.4 nmol/l) were comparable in age (~53 years) and sex distribution (~49% female). Most participants with elevated Lp(a) were previously unaware of their levels; 30% were referred to specialists, and ~40% reported concern or anxiety. Combined cardiovascular endpoints (CHD, stroke, heart failure, PAD, carotid stenosis, AF) occurred significantly more often in Gr2 (p<0.001), despite similar CVRF profiles, except for higher baseline LDL-C in Gr2 (p<0.001). Hypertension (61%) and physical inactivity (57%) were the most prevalent CVRFs. Personalized prevention measures led to significant improvements in blood pressure, LDL-C, smoking, physical activity, and weight in both groups. Lipid-lowering therapy improved markedly in Gr2 (12% to 23%).

**Conclusions:**

Elevated Lp(a) is associated with a significantly higher rate of cardiovascular events, independent of traditional CVRFs. This confirms a causal role of Lp(a) in CV morbidity. Personalized written prevention recommendations improved CVRFs across both groups, though further optimization is needed. Notably, elevated Lp(a) also imposes a psychosocial burden, underlining the need for enhanced education, counseling, and clinical pathways.

## Introduction

Cardiovascular diseases remain the leading cause of death worldwide, with cases continuing to rise in Germany [1]. This is due to insufficient control of identified cardiovascular risk factors (CVRF) [2]. The ELITE study in north-west Germany is prospectively collecting and analyzing extensive data on cardiovascular and cerebrovascular risk factors. In addition to recording classic risk parameters such as blood pressure, weight, glucose and lipid metabolism parameters, uric acid, kidney function and smoking, detailed surveys are also conducted on physical activity, dietary habits, depressive symptoms, mental stress, well-being and medication use. Regular measurements of lipoprotein (a) are also carried out. After the initial examination, all participants receive detailed written information about their individual profile with specific suggestions for better cardiovascular prevention. Regular follow-up examinations with regular information about the individual risk profile are intended to improve the control of risk factors and thus prevent the development of vascular events. The follow-up examinations record the recommendations that have been successfully implemented and evaluate the individual reasons why proposed preventive measures have not been implemented.

This study evaluated data on elevated lipoprotein (a) [Lp(a)]. This is a particular focus of this study, as no data is available for north-western Germany. Lipoprotein (a) [Lp(a)] has been proven in numerous studies to be an independent cardiovascular risk factor [3, 4]. The updated guidelines assume that an Lp(a) level above 105 nmol/L is a relevant risk factor [5]. The relevance of recurrent Lp(a) measurements is being discussed among experts. Surma et al. discussed the fluctuation especially during hospital admission. Acute phase reaction and statin therapy can be causative for variation. This can be relevant in therapy decisions and further treatment [6]. There is currently no direct drug therapy for elevated Lp(a), however it is currently being developed [7]. These include Pelacarsen, an antisense oligonucleotide drug, Olpasiran, a small interfering RNA, and Muvalaplin, a small molecule drug, which are currently being investigated in studies [8, 9]. Lifestyle modifications such as diet or physical exercise do not significantly influence Lp(a) levels [10]. Currently, the only treatment available is lipid apheresis, which can effectively lower Lp(a) levels and reduce the risk of serious cardiovascular events. However until now, there are no randomized controlled studies showing the benefits of Lp(a) apheresis. Only the Pro(a)LiFe study, a prospective observational study showed a significant reduction in cardiovascular events in patients with elevated Lp(a) which have undergone lipid apheresis [11]. However, in Germany, apheresis treatment is reserved for high-risk patients as part of secondary prevention [12].

The purpose of this study is to record and treat the frequency of other CVRF that can further increase the risk posed by Lp(a). This study also analyses changes in CVRF and the occurrence of cardiovascular events in individuals with and without elevated Lp(a) over a period of almost five years. Of particular interest in this paper is the care situation of participants with elevated Lp(a) and the associated psychosocial aspects [13, 14].

## Methods

### Study design

The ELITE (Ernährung (nutrition), Lebensstil (lifestyle) und individuelle Information (individual information) zur Verhinderung von Herzinfarkt, Schlaganfall und Demenz (for prevention of heart attack, stroke and dementia)) study is a prospective, interventional cohort study with a five-year follow-up and 5.726 participants aged 16 years and older. In total, 812 individuals participated only in the initial examination. 4.914 individuals agreed to participate in a long-term study. Of these, 832 individuals discontinued the study. 4.082 individuals were available for evaluation. Four patients could not be included in the final evaluation because they did not undergo lipoprotein (a) determination during the initial presentation and died before a follow-up visit. The mean follow-up period was 4.4 years.

Two groups were compared: Group 1: Lp(a) values < 75 nmol/L and Group 2: Lp(a) values > 75 nmol/L. This was chosen because our laboratory reports values > 75 nmol/l as elevated and the participants were informed of this.

The study design was approved by the Ethics Committee of the University of Göttingen (application number 34/6/14) based on the ICH-GCP guidelines. Further key findings have already been published [15–19]. Volunteers were recruited in northern Germany through invitations to sport and social clubs, government agencies, companies and public institutions, as well as through publicity in newspaper advertisements, flyers and presentations. Part of the study involves creating an annual cardiovascular risk profile for participants based on sports and nutrition data, laboratory values [including LDL, HDL, Lp(a) and HbA1c], medical histories and blood pressure data, as well as a brain performance test, and then making recommendations in line with guidelines. Participants with elevated Lp(a)-levels were given detailed information about the findings and advised on the importance of controlling all other risk factors, especially LDL cholesterol. Four people did not have their Lp(a)-levels measured at the start of the study and died before the second visit, so no Lp(a) values were available. The effectiveness of the recommendations is re-evaluated in annual follow-ups. Participants with elevated Lp(a) completed a supplementary questionnaire after five years, which asked about their care situation and psychosocial aspects. These are based on validated questionnaires for assessing psychological stress and depressive symptoms. The evaluation of possible depression or depressive tendencies was analyzed using the BDI II test. Here, a profile with a point score was created based on 21 questions about mental health, and patients were categorized accordingly. All results were included in the evaluation in addition to the known cardiovascular risk factors. Cardiovascular events and overall mortality were evaluated after 4.4 years.

### Data analysis

#### Cardiovascular Risk Factors (CVRF)

For the purposes of this study, CVRF are defined as: smoking, diabetes mellitus, LDL cholesterol greater than 130 mg/dl, blood pressure above 140/90 mmHg or, in the case of prior antihypertensive treatment, a BMI greater than 30 kg/m², and nicotine abuse.

#### Physical Activity (PA)

Sports habits were classified based on the answer to the multiple-choice question in the questionnaire ‘Do you participate in sports?’

– Regular physical activity = ‘yes, daily’ and/or ‘2–3 times a week’
– Moderate amount of sport/physical activity = ‘once a week’ and ‘every two weeks’
– Rarely exercise/engage in physical activity = ‘once a month’ and ‘less often’

Regular physical activity was considered a therapeutic goal and subsequently classified as sufficient.

### Psychological stress

The question ‘How often do you feel exposed to stressful situations in your everyday life?’ from the questionnaire was classified as stress if the answer was ‘2–3 times a week’ or ‘yes, every day’.

### Symptoms of depression

The BDI-II (Beck, Ward, Mendelson, Mock and Erbaugh; 1961) was used to screen for depression; a score of 0 to 8 points is defined as normal.

### Nicotine consumption

All patients who smoke, regardless of quantity and frequency.

### Diabetes mellitus

All participants who have known type I or type II diabetes mellitus, who are taking antidiabetic medication, or whose haemoglobin A1c (HbA1c) is ≥ 6.5%.

### Hypertensive patients

All participants who have high blood pressure (140/90mmHg) or are taking antihypertensive medication.

### Normotensive patients

All participants who have normal blood pressure readings and are not taking blood pressure medication or have a history of high blood pressure.

### Lp(a)-Level

Lp(a)-levels were measured using the Tina-quant^®^ Lipoprotein (a) Gen. 2 test from Roche:

Normal: <75 nmol/l.

### Cardiovascular endpoints

The primary combined cardiovascular endpoint (CVE) was defined as the occurrence of coronary heart disease (CHD) with or without myocardial infarction, stroke, carotid stenosis, atrial fibrillation or peripheral artery disease (PAD). The endpoints were primarily recorded in letters from hospitals or general practitioners and based on information provided by participants and their relatives.

There was no change in drug therapy within the study team—only a recommendation to the family doctor to optimize it specifically if necessary. Initially, the participants were asked about any events that had occurred or hospital or specialist treatments during their visits. The events were then evaluated as described, primarily on the basis of letters from clinics, specialists, and general practitioners. Since most events were treated in clinics, the clinical reports were mainly used as a basis, which means a high level of medical expertise. In addition, specialist reports and, where applicable, GP reports were also used. This means that over 90% of all events could be evaluated with a high level of medical expertise. Self-reported information from participants or relatives was only obtained in rare exceptional cases, e.g. if the patient was no longer able to attend visits or had died.

### Statistic

The statistical analysis was performed using the IBM SPSS Statistics software package, version 30. For all metric variables collected, descriptive statistics such as mean with standard error, minimum, maximum and standard deviation were calculated at the beginning of the analysis. Furthermore, the data were presented graphically in the form of histograms. The t-test for unpaired samples was used to compare the mean values of two independent groups. To compare two paired samples, e.g. the same test subjects for admission and visit 1, the t-test for dependent samples was used. For the data from categorical variables, frequency tables and corresponding bar charts were first created. This information was then summarized in the form of cross tables. The Pearson chi-square test was used to check for possible correlations or dependencies between categorical variables at the same point in time. For paired samples of categorical variables, the McNemer test was used to determine significant changes over time. Multiple logistic regression was applied in order to evaluate the effects of Lp(a) on cardiovascular endpoints by taking into account also other covariates (confounder). The usual significance level of α = 0.05 was chosen for all statistical tests. Thus, for tests that yielded a p-value less than 0.05, the null hypothesis was rejected, and the alternative hypothesis was accepted.

## Results

A total of 4.078 study participants were evaluated. Of these, 3.233 participants had normal Lp(a) levels (< 75 nmol/l, Gr1) and 845 participants had elevated Lp(a)-levels (> 75nmol/l, Gr2). Thus, 20.7% of participants had elevated Lp(a) levels. There were no differences in age or gender between the two groups (women: Gr1: 49.0%, Gr2: 49.4%, (*p* = 0.817); age: Gr1: 52.9 years, Gr2: 53.1 years, (*p* = 0.817)). The mean Lp(a) level was 16.5 nmol/l in Gr1 and 154.6 nmol/l in Gr2. In their personal medical history, participants in Gr2 reported significantly more frequently known lipid metabolism disorders (Gr1: 16%, Gr. 2: 24%, *p* < 0.001). Atrial fibrillation was more common in Gr1: 4.0% than in Gr2: 2.3% (*p* < 0.016). There were no other differences in CVRF or previous non-cardiac illnesses in the medical history. The frequency of previous cardiovascular and cerebrovascular diseases was low, ranging between 1.4% and 5.4%, with no significant difference between the groups. In the family history, only the number of coronary heart disease cases was significantly higher in group 2 (Gr1: 22.1%, Gr2: 27.5%, *p* < 0.001). There were no other differences in the family history.

**Fig. 1:**
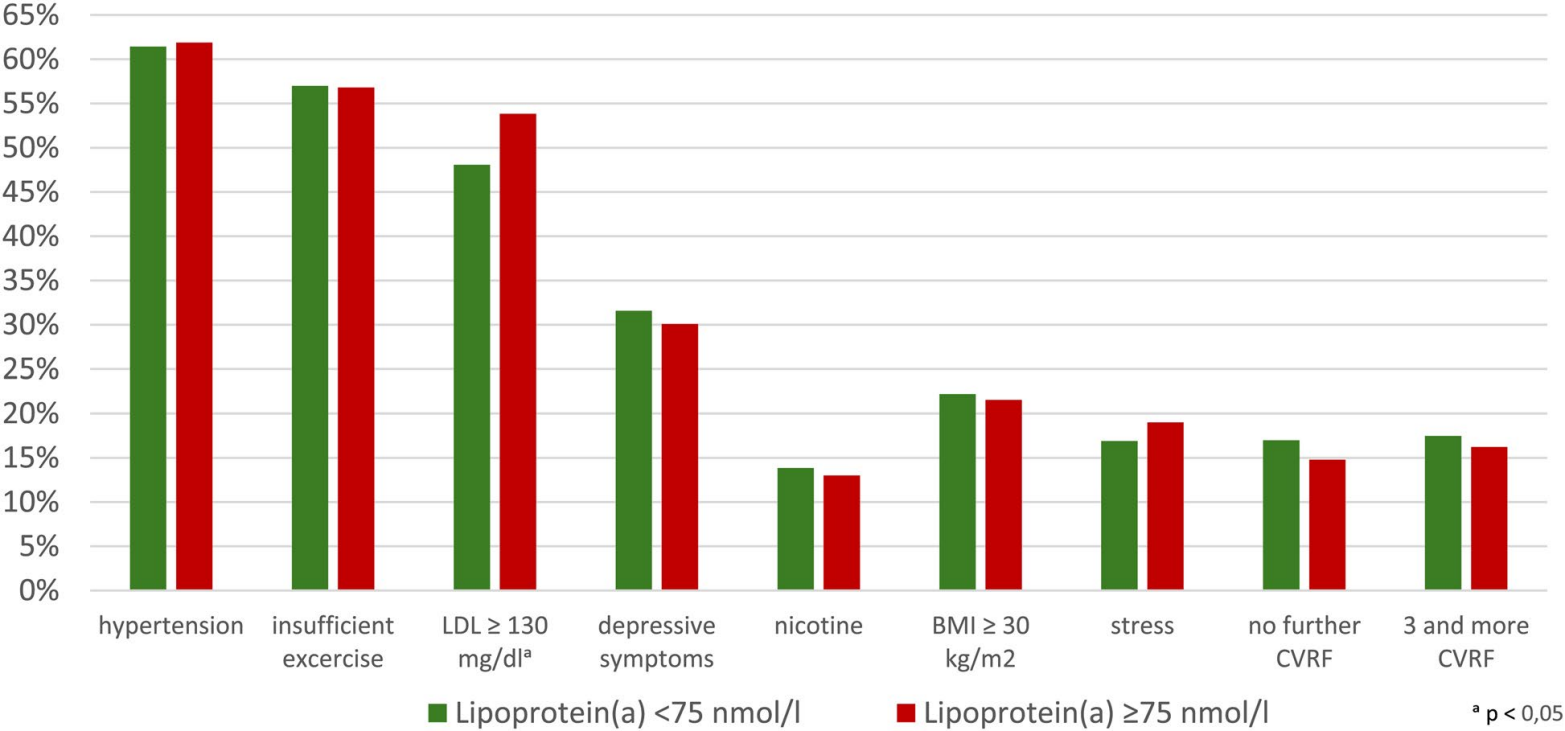
Frequency of risk factors in Lp(a) Group at admission in comparison between Lp(a) groups

Figure 1 shows the frequency of CVRF in both groups at the start of the study. The most common CVRF in both groups was arterial hypertension, with no significant difference between the two groups at admission. The only significant difference was in the frequency of high LDL cholesterol (48.1% in Gr1; 53.9% in Gr2 (*p* = 0.003)). Table 1 also shows the mean values of individual CVRF. There was a significant difference in mean LDL cholesterol (Gr1: 130.6 mg/dl, Gr2: 135.4 mg/dl, *p* < 0.001). CVRF were therefore similarly prevalent in both groups. Since several risk factors have a synergistic effect, the frequency of risk factors (hypertension, BMI > 30 kg/m2, LDL-C > 130 mg/dl, nicotine, diabetes) was evaluated per participant. Only 17% of participants in Gr1 had no risk factors at the start of the study, compared with 14.8% in Gr2 (Table 2). On the other hand, 17.5% of participants in Gr1 had three or more risk factors, compared with 16.2% in Gr2. In Gr1, 16.9% reported ‘daily negative stress’, compared with 19% in Gr2 (*p* = 0.152). Depressive symptoms were initially comparable in both groups (31.6% vs. 30.1%, *p* = 0.447).

**Table 1:** Frequency of risk factors (RF) in Lp(a) groups at baseline in comparison between the two groups. *Values in bold are statistically significant

	Lipoprotein(a) < 75nmol/l	Lipoprotein(a) ≥ 75nmol/l	Sign.
	*n* (%)	*n* (%)	
hypertension (total)	1986 (61,4%)	523 (61,9%)	0,812
insufficient exercise	852 (57,0%)	221 (56,8%)	0,936
LDL ≥ 130mmol/l	1554 (48,1%)	455 (53,8%)	**0**,**003**
BDI	910 (31,6%)	219 (30,1%)	0,447
Nicotine	448 (13,9%)	110 (13,0%)	0,574
BMI ≥ 30 kg/m2	718 (22,2%)	182 (21,5%)	0,709
Diabetes	201 (6,2%)	46 (5,4%)	0,466
stress daily	543 (16,9%)	160 (19,0%)	0,152
no further CVRF	549 (17,0%)	125 (14,8%)	**< 0**,**001**
3 and more CVRF	565 (17,5%)	137 (16,2%)	**< 0**,**001**

**Table 2:** Frequency of risk factors (RF) in Lp(a) groups at baseline and at the end of the study. *Values in bold are statistically significant

	Lipoprotein(a) < 75nmol/l			Lipoprotein(a) ≥ 75nmol/l		
	Admission *n*(%)	final *n*(%)	Sign.	Admission *n*(%)	final *n* (%)	Sign.
BMI ≥ 30 kg/m2	718 (22,2)	650 (20,1)	**< 0**,**001**	182 (21,5)	175 (20,7)	0,483
hypertension (total)	1986 (61,4)	1996 (61,7)	0,719	523 (61,9)	520 (61,5)	0,888
insufficient exercise	1608 (56,3)	1493 (52,3)	**< 0**,**001**	409 (56,6)	394 (54,5)	0,293
BDI	910 (31,6)	959 (33,3)	0,077	219 (30,1)	256 (35,2)	**0**,**008**
Nicotine	446 (13,8)	345 (10,8)	**< 0**,**001**	108 (12,8)	85 (10,2)	**< 0**,**001**
Diabetes	201 (6,2)	202 (6,2)	1,00	46 (5,4)	56 (6,6)	0,100
stress daily	543 (16,9)	506 (15,7)	0,126	160 (19,0)	138 (16,4)	0,086
LDL ≥ 130 mg/dl	1554 (48,1)	1316 (40,7)	**< 0**,**001**	455 (53,8)	355 (42,0)	**< 0**,**001**
no further CVRF	549 (17,0)	664 (20,5)	**< 0**,**001**	125 (14,8)	160 (18,9)	**0**,**002**
3 and more CVRF	656 (17,5)	433 (13,4)	**< 0**,**001**	137 (16,2)	107 (12,7)	**0**,**004**

At the end of the observation period, improvements were found in most CVRFs. This is also shown in Tables 1 and 2. Significant improvements occurred in Gr1 and Gr2 in the frequency and mean value of LDL cholesterol (all *p* < 0.001). LDL-C was reduced from 130.5 mg/dl to 127.7 mg/dl in Gr1 and from 135.4 mg/dl to 129.1 mg/dl in Gr2. The initial average Lp(a) value was 16.5 nmol/L in GR1 and 154.1 nmol/L in GR2. Over the course of the study, both groups recorded a significant increase to 18.8 nmol/l and 164.2 nmol/l, respectively (*p* < 0.001).

The Lp(a)-levels of women and men were not different in either group at the start. However, they rose more sharply in women than in men. The final values were significantly higher in women than in men in both groups (*p* = 0.006 and *p* = 0.048, respectively) Tables 3 and 4.

**Table 3:** Lipoprotein(a) distribution. *Values in bold are statistically significant

	**admission**	**final**	
	**Mean (nmol/l)**	**Mean (nmol/l)**	**Sign.**
total	45,12	50,03	**< 0**,**001**
Lp(a) < 75 nmol/l	16,52	18,85	**< 0**,**001**
Lp(a) < 75 nmol/l	154,06	164,24	**< 0**,**001**
Lp(a) 75–120 nmol/l	97,12	108,59	**< 0**,**001**
Lp(a) > 120 nmol/l	186,94	194,56	**< 0**,**001**

**Table 4:** Lipoprotein(a) distribution according to gender

	**admission**			**final**		
	**male**	**female**		**male**	**female**	
	**mean (nmol/l)**	**mean (nmol/l)**	**Sign.**	**mean (nmol/l)**	**mean (nmol/l)**	**Sign.**
total	44	46,28	0,265	47,48	52,76	**0**,**044**
Lp(a) < 75 nmol/l	16,16	16,9	0,166	17,56	20,25	**0**,**006**
Lp(a) ≥ 75 nmol/l	151,65	157,62	0,199	158,29	170,51	**0**,**048**

Furthermore, the influence of statins is listed in Table 5. At the beginning and end of the study, participants who had been taking statins prior to the study had significantly higher Lp(a)-levels than participants who had not been treated with statins (*p* < 0.001). In group 1, however, there was no significant difference at the beginning (*p* = 0.44) or at the end (*p* = 0.229) of the study.

**Table 5:** Lipoprotein(a) Levels with and without lipid-lowering-therapy (LLT). *Values in bold are statistically significant

	**admission**			**final**		
	**without LLT**	**with LLT**		**without LLT**	**with LLT**	
	**mean (nmol/l)**	**mean (nmol/l)**	**Sign.**	**mean (nmol/l)**	**mean (nmol/l)**	**Sign.**
total	43,47	60,93	**< 0**,**001**	46,48	74,34	**< 0**,**001**
Lp(a) < 75 nmol/l	16,39	17,76	0,144	18,63	20,53	0,229
Lp(a) ≥ 75 nmol/l	150,9	181,87	**< 0**,**001**	158,14	190,49	**< 0**,**001**

*LLT = lipid-lowering-therapy

The Lp(a)-levels of women and men were not different in either group at the beginning. However, they rose more sharply in women than in men. The final values were significantly higher in women than in men in both groups (*p* = 0.006 and *p* = 0.048).

Based on the recommendations, this was also largely due to a higher rate of lipid-lowering drug prescriptions (almost exclusively statins). The number of people taking lipid-lowering drugs rose from 8.8% to 12.5% in Gr1 (*p* = 0.005) and from 12.0% to 23% in Gr2 (*p* < 0.001). At baseline, 8.6% of Gr1 Lp(a) group were taking statins, while 0.2% were taking ezetimibe. In contrast, 11.6% of people in Gr2 initially took statins and 0.4% took ezetimibe. By the end of the study, the proportion of people in Gr1 taking statins had risen to 11.6%, while the proportion taking ezetimibe had risen to 0.4%. In the Gr2, prescriptions for statins increased to 17.9% and prescriptions for ezetimibe increased to 1.6%. Only one patient received a PCSK9 inhibitor. Under statin therapy, Lp(a) increased by an average of 5,3 nmol/l (Admission: 69,0 nmol/l, Completion: 74,3nmol/l, *p* < 0.001). The group without lipid-modifying therapy also experienced a significant increase (Admission: 42,2 nmol/l, Completion: 46,5 nmol/l, *p* < 0.001).

Nicotine abuse and the average reduction in blood pressure among hypertensive patients also improved in both groups. Only in Gr1 were there significant improvements in weight and physical activity. The significant reduction in nicotine abuse in both groups was particularly encouraging (*p* < 0.001).

While no significant changes occurred in Gr1 with regard to ‘daily negative stress’, there was a tendency towards improvement in Gr2 from 19.0% to 16.4% (*p* = 0.086). Depressive symptoms in BDI II were similarly frequent at the start. They increased slightly in both groups (Gr1 *p* = 0.077, Gr2 *p* = 0.008) (Table 1). Overall, the number of prescriptions for psychotropic drugs was 7%, with no significant difference between the two Lp(a) groups. There was also no correlation with socioeconomic status, school education, vocational training, or profession, nor with gender or age.

The extent of the improvements described was also evident in the number of CVRFs per person. It was encouraging that at the end of the study, the number of people without risk factors increased significantly in both groups: in Gr1 from 16% to 20.5%, and in Gr2 from 14.9% to 19.0% (*p* < 0.001).

Accordingly, the number of participants with 3 or more risk factors fell from 17.6% to 13.5% in Gr1 and from 16.1% to 12.7% in Gr2 (*p* < 0.001).

**Fig. 2:**
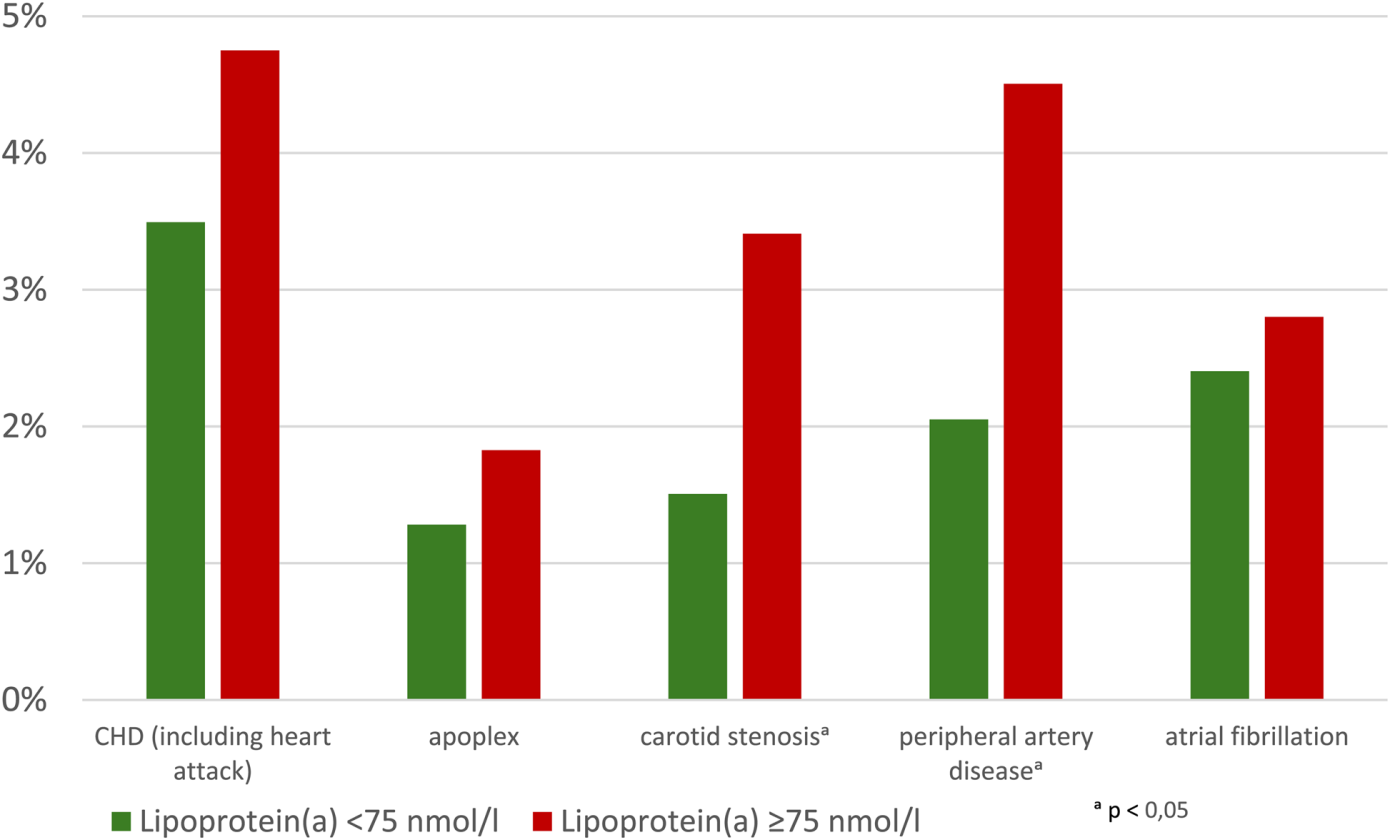
Distribution of cardiovascular Endpointsin both groups

Figure 2; Table 6 shows the frequency of CVE in both groups. The primary endpoints were CHD with and without myocardial infarction, heart failure, stroke, PAD, carotid stenosis and atrial fibrillation. CVE occurred significantly more frequently in Gr2 with elevated Lp(a) (*p* < 0.001). This applied to both CVE groups with and without mortality. This is also confirmed by multiple logistic regression, when controlling for possible confounders (Table 7). Overall mortality, cardiovascular mortality and non-cardiovascular mortality did not differ. For the individual endpoints, the frequency of carotid stenosis and PAD was significantly higher in Gr2. There was a slight trend towards CHD, but this was not significant. However, there was a significantly increased number of CHD cases in Lp(a) values above 120 nmol/l compared to individuals with Lp(a) values < 120 nmol/l (*p* = 0.03). A total of 531 CV endpoints occurred in 426 individuals. This is equivalent to a frequency of 1.22% in Gr1 and 1.31% in Gr2.

**Table 6:** Endpoint distribution. *Values in bold are statistically significant

	**Lipoprotein(a) < 75nmol/l**		**Lipoprotein(a) ≥ 75nmol/l**		**Sign.**
	n (%)		n (%)		
Number of patients with CVEP* (without mortality)	311 (10,0)		115 (14,0)		**< 0**,**001**
Number of patients with CVEP (incl. mortality)	369 (11,4)		128 (15,1)		**0**,**003**
Total mortality	113 (3,5)		24 (2,8)		0,347
Of which cardiovascular mortality	65 (57,0)		14 (58,3)		0,546
CHD (including heart attack)	109 (3,5)		39 (4,8)		0,092
apoplex	40 (1,3)		15 (1,8)		0,236
carotid stenosis	47 (1,5)		28 (3,4)		**< 0**,**001**
peripheral artery disease	64 (2,1)		37 (4,5)		**< 0**,**001**
atrial fibrillation	75 (2,4)		23 (2,8)		0,515

*CVEP= cardiovascular endpoint, Number of patients with CVEP: All patients who suffered one or more CVEP were counted here

**Table 7:** Logistic regression results. *Values in bold are statistically significant

**Variable**	**Regression coefficient B**	**Standard Error**	**OR (Exp(B))**	**Sign.**
Age	0,087	0,005	1,091	**< 0.001**
Gender (m)	0,348	0,108	0,706	**0.001**
Nicotine	0,521	0,163	1,684	**0.001**
Hypertension	0,537	0,14	1,71	**< 0.001**
BMI ≥ 30	0,284	0,119	1,329	**0.017**
LDL-C	0,004	0,002	0,996	**0.009**
Lp(a)-category	0,453	0,122	1,574	**< 0.001**

*CVEP Cardiovascular endpoint, Number of patients with CVEP: All patients who suffered one or more CVEP were counted here

### Subjective assessment by participants and awareness

Overall, 69.6% of participants reported that they were able to implement measures and recommendations, with no significant differences between the Lp(a) groups. Recommendations on diet were by far the most frequently implemented, followed by increasing physical activity. Significantly more participants with elevated Lp(a) reported a reduction in lipids.

The additional questionnaire on awareness was completed by 57.2% (*n* = 481) of participants with elevated Lp(a). Of these, 67.4% of participants stated that they had already been informed about the risks of Lp(a). 22.0% reported that they had been informed by the ELITE study, so it can be assumed that the majority (approx. 55%) of participants were not aware of Lp(a) prior to the study. Just under half of the participants had discussed the values with their general practitioner, of whom only 29.1% were referred to a specialist. The most common referral was to a cardiologist (59.5%). It is interesting to note that awareness of Lp(a) often led to lifestyle changes (reported by 37.8%), while less frequently resulted in a change in medication (reported by 13.7%). In line with this, the elevated Lp(a) value was associated with increased self-reported anxiety or concern in 39.3% of participants.

## Discussion

The data from the ELITE study presented here reveal the distribution of Lp(a) in the north-western German population for the first time. At just under 21%, the population studied here is in line with other northern European regions [13]. The increasing number of CVE in recent years highlights the urgent need for a comprehensive prevention strategy. Data already published from the ELITE study show that, in addition to elevated Lp(a), modifiable cardiovascular risk factors are disproportionately prevalent in the risk group and that adequate prevention is often inadequately implemented [18]. The five-year evaluation now available allows the successful outcomes of the intervention and the distribution of endpoints to be analyzed for the first time. The main findings from this analysis were as follows: (1) The number of CVEs (CHD with/without MI, stroke, PAD, carotin stenosis, AF) were significantly more frequent with elevated Lp(a). (2) without significant differences in the expression of the other risk factors in both groups, this difference is mainly due to the elevated Lp(a). (3) the majority of those affected were unaware of their elevated Lp(a) (4) Despite repeated risk communication and education, only a minority of at-risk patients were referred to a specialist. (5) 40% of those affected reported anxiety or worry about their elevated Lp(a), making Lp(a) a previously unknown psychological risk factor.

CVEs with and without mortality occurred significantly more frequently in patients with elevated Lp(a) (*p* < 0.001). Total mortality and cardiovascular mortality did not differ. The collective was relatively young on average and the follow-up period was only 4.4 years. This is likely to be the reason for the lack of difference in overall and cardiovascular mortality. The most common CVD in both groups was CHD, without significant difference. Due to the existing trends, with a percentage-wise but not significant overrepresentation of newly occurring CHD, the evaluation was performed according to Lp(a) concentration. This confirmed that a significantly elevated Lp(a) level of >120 nmol/l is associated with a significantly higher incidence of new CHD [Lp(a) < 120 nmol/l: 4.0%, Lp(a) >120 nmol/l: 6.1%, *p* = 0.030]. The differences in heart failure, stroke and atrial fibrillation were not significant. In contrast, carotid stenosis and PAD were significantly more common in patients with elevated Lp(a). It is possible that PAD and carotid stenosis manifest themselves before the onset of ischemic events such as stroke or CHD. Only LDL-C was significantly higher upon admission to Gr2, which is partly due to the LDL-C content of Lp(a) [20]. However, LDL-C fell significantly in both groups, which can be explained by a significant increase in the prescription of lipid-lowering drugs, especially statins, with the increase in prescriptions of lipid-lowering drugs being significantly higher in Gr2. Conversely, a significant increase in Lp(a) levels was observed in patients taking statins. A smaller but still significant increase in Lp(a) levels was also observed in the group not receiving statin therapy. Overall, an increase in Lp(a) levels was evident in both groups and in all subgroups. Significant increases in Lp(a) were also observed when the data were broken down by gender. One reason for this could be that people with elevated Lp(a) were made very aware of the significance of elevated LDL cholesterol and were urgently advised to see a doctor. Early LDL-C reduction in Lp(a) patients has been shown by Ferrence et al. to be a potential strategy to reduce overall risk in an analysis of 445,765 patients from the UK Biobank [21]. Overall, the relevance of under-treatment of hypercholesterolemia is clear. This was also confirmed in the SANTORINI study (Treatment of High and Very High riSk Dyslipidemic pAtients for the PreveNTion of CardiOvasculaR Events). Around 2,000 patients (average age 66) from Germany were analysed. Overall, 80% of patients with high risk and very high risk did not achieve the defined target value. At the start of the study, one fifth of patients did not take lipid-lowering drugs [22]. In our cohort, statin therapy was significantly increased through intensive written recommendations, although it remains alarmingly rare at approximately 20% in the pathological Lp(a) group. One limitation here is that participants were referred to their general practitioner for further treatment with the recommendations. Even though the majority of patients with hyperlipoproteinemia reported having discussed the findings with their GP, the reasons preventing lipid-lowering therapy cannot be fully identified. It remains to be postulated that outpatient management of LDL cholesterol in patients with hyperlipoproteinemia is inadequate.

Of course, the care provided is completely unacceptable. However, this applies equally to the whole of Germany and Europe, as recent data and also surveys from Germany show. In addition, the study was conducted in a predominantly rural region in north-west Germany with a low density of doctors and relatively long distances.

The same applies to further lipid modifying therapies. Only one person was treated with an PCSK9 Inhibitor. Patients with apheresis were not included in the study. These comparatively low figures are most likely due to cost and structural limitations in Germany as only specific patients were eligible for treatment, and initially only certain specialists were permitted to administer these therapies.

The risk of elevated Lp(a) in relation to LDL-C was clearly illustrated by an analysis of the Copenhagen General Population Study. Hedegaard et al. attempted to make the risk of hyperlipoproteinemia more tangible in comparison to familial hypercholesterolemia. An Lp(a) level above 150 nmol/L is equivalent to the risk of clinically diagnosed familial hypercholesterolemia. At plasma levels above 180 nmol/L, participants had an identically high risk of myocardial infarction as genetically diagnosed familial hypercholesterolemia [23].

Overall, the repeated prevention recommendations in ELITE significantly improved the number of risk factors per person in both groups. However, the extent of the reduction is not yet sufficient to achieve a sustained reduction in cardiovascular events. Ultimately, despite improved risk factors, Gr2 with elevated Lp(a) showed significantly more cardiovascular events after 4.4 years.

Strict and early control of all CVRFs is therefore essential. In our cohort, written prevention recommendations improved mean blood pressure in either group, even though the combination of high blood pressure and elevated Lp(a) has also been shown to dramatically increase cardiovascular risk. In a prospective study spanning almost 14 years, Liu et al. showed that patients with elevated Lp(a) and additional hypertension had the highest risk of cardiovascular disease compared to patients with isolated hypertension or isolated hyperlipoproteinemia. The authors therefore hypothesized that hypertension causes vascular damage, allowing Lp(a) to exert its proatherogenic properties [24]. “The Mesa” study also demonstrates this impressively. In this prospective study by Rikhi et al., the cardiovascular risk of isolated hyperlipoproteinemia was not increased compared to a reference group, but only increased significantly when high blood pressure occurred simultaneously [25].

In particular, a lack of physical exercise is a missed opportunity for evidence-based preventive medicine. Although repetitive written information has significantly increased physical activity, the potential of sport is still far from being fully exploited. It has been clearly and repeatedly proven that physical activity at the recommended level leads to a better cardiovascular risk profile [26, 27]. In this cohort, adequate physical activity was also associated with better blood pressure, lower BMI, healthier diet and less psychological stress than in physically inactive individuals [28]. Despite this, only about 43% of the cohort were physically active enough.

The 3 F studies already demonstrated that regular exercise can safely optimise blood pressure profiles. In this study, patients were introduced to exercise through regular physical activity as part of a modified football training program, and their vital signs were evaluated during training and in everyday life [29, 30].

There is also clear room for improvement in terms of education and awareness. The majority of participants with elevated Lp(a) were unaware of its pathogenicity, and only about 30% were referred by their general practitioner to specialists. For the vast majority of participants with elevated Lp(a), the pathological finding had no impact on their medication or lifestyle. This leads to the subjective assessment of those affected that they do not feel adequately cared for by their doctors, which in turn leads to anxiety in almost 40% of participants. However, some results regarding psychosocial issues have not yet been fully evaluated. Since the Elite Study placed particular emphasis on psychosocial factors, further analyses of the influence of these factors will follow based on the data collected here.

The significant reduction in nicotine abuse in both groups is encouraging. The literature shows that smoking in combination with elevated Lp(a) increases cardiovascular risk [31, 32]. This group therefore benefits greatly from giving up nicotine. The success of the therapy is attributed to intensive education and regular reminders about the significantly increased risk of cardiovascular disease. Other studies have also shown that the prevention of cardiovascular disease is not being implemented optimally. In a systematic review Álvarez-Bueno et al. found that the effectiveness of primary prevention of cardiovascular disease in primary care has only limited to moderate success [33]. In addition, a study in the USA showed that around half of doctors do not follow guidelines for the prevention of cardiovascular disease in 50% of cases [34].

The debate surrounding adequate, comprehensive cardiovascular prevention in Germany (similar to various oncological diseases) is currently louder than ever. The DANCAVAS study investigated whether cardiovascular screening tests can reliably and effectively identify people with heart disease. To this end, 46,000 people in Denmark between the ages of 65 and 75 with no previous cardiovascular disease were randomly assigned (2:1) to a screening group (cardio CT, cholesterol, blood sugar) and a non-screening group. In a relatively short observation period of approximately 5 years, the mortality risk in the screening group was reduced (from 13.1% to 12.6%) [35]. It is hoped that Lp(a) will be included in future preventive studies. However, this poses a problem, as established risk scores do not currently include Lp(a). In this regard, Nurmohamed et al. conducted an evaluation in which they included Lp(a) in the SMART Score and SCORE. When Lp(a) was taken into account, 31% of primary prevention patients and 62.5% of secondary prevention patients with elevated Lp(a) had to be reclassified into a higher risk category [36]. Again, the authors suggest routine testing.

Although the data available from the ELITE study demonstrate the effectiveness and success of regular, individual counselling in controlling CVRF, they also highlight major shortcomings in outpatient care. This is particularly relevant for the patient group with elevated Lp(a), which is already an intrinsic high-risk group. The high-risk group of patients with elevated Lp(a) requires even more attention with regard to the control of all modifiable CVRF. The most prominent example here is the lack of adherence to lipid-lowering therapy as also shown in EUROASPIRE [37]. These results give cause not only to educate patients about the benefits and necessity of treatment with lipid lowering therapy, but also to seek dialogue with the treatment general practitioners in order to ensure adequate therapy. This has been done in the ELITE study.

In the ÄSP study, Schneider et al. already investigated cardiovascular prevention work carried out by general practitioners in Germany. This revealed concerning deficits in cardiovascular risk communication and prevention. The main reason given is, in the eyes of the general practitioners, the intervention is unlikely to be successful. The data from the ELITE study prove the opposite. This makes it all the more important to communicate the successes of adequate risk prevention in the outpatient sector as well. Good risk communication works [38].

### Limitations and strengths

One limitation of this registry study is the lack of a randomized control group. Majority of the data collected is based on information provided by the participants, which means that the accuracy of the information cannot be verified. In any case, the majority of data were collected primarily through letters from clinics, specialists and general practitioners. This ensured a high level of expertise regarding new diagnoses and changes in patients’ health. However, this is offset by the fact that all participants were regularly examined and interviewed in person. Where possible, the reasons for changes in responses were also investigated. The close contact and personal visits provide a relative assurance that the information provided by the participants is truthful. For the accurate recording of cardiovascular events, we relied on the corresponding findings of the treating physicians. Therefore, in the event of missing data, research was carried out at hospitals, with physicians and also with the participants or their relatives. The strengths of this study lie in the systematic and thorough recording of all CVRF and the repeated personal visits. In addition, the personal situation and lifestyle (physical activity, stress, depression, well-being) were also recorded. A particular strength is the personal visits and conversations, including written recommendations.

## Conclusion

Overall, this study also confirms the high prevalence and cardiovascular risk posed by Lp(a) in a rural population and identifies three major problem areas: lack of adherence to outpatient guidelines, lack of patient awareness and fear among affected patients resulting from elevated Lp(a)-levels and medical neglect.

Intensified written recommendations and recurring information about risk factors lead to an improvement in CVRF in many individuals. However, the extent of the improvements is insufficient. This shows that outpatient cardiovascular prevention in Germany is not adequate. There is a lack of enthusiasm, particularly in outpatient care, for engaging in what is often lengthy and seemingly inefficient risk communication with patients.

This also applies in particular to the high-risk group with high Lp(a) levels. Comprehensive screening and education for CVRF is an absolute must, especially for the very common risk factor Lp(a). Demographic trends give rise to concerns that capacities for the prevention of cardiovascular diseases will not be improved. This also applies to Lp(a) elevation. More specialist training is needed in the outpatient sector in order to exploit the full preventive potential, especially when drug therapy is available. The crucial question will once again be how we can quickly transfer scientific findings to rural areas. Once the medication against elevated Lp(a) has been approved, an urgent ethical question will be whether and when it should also be used in primary prevention in high-risk patients, especially as elevated Lp(a) also appears to lead to psychological stress.

## Data Availability

No datasets were generated or analysed during the current study.

## Abbreviations

AF: Atrial Fibrillation
CAD: Coronary Artery Disease
CVE: Cardiovascular Event
CVRF: Cardiovascular Risk Factor
ELITE: Ernährung, Lebensstil und individuelle Information zur Verhinderung von Herzinfarkt, Schlaganfall und Demenz (german for: Nutrition, lifestyle and individualized information for the prevention of heart attack, stroke and dementia)
MI: Myocardial Infarction
PAD: Peripheral Arterial Disease
PA: Physical Activity
